# Abstract Word Definition in Patients with Amnestic Mild Cognitive Impairment

**DOI:** 10.1155/2015/580246

**Published:** 2015-08-09

**Authors:** Soo Ryon Kim, SangYun Kim, Min Jae Baek, HyangHee Kim

**Affiliations:** ^1^Graduate Program in Speech and Language Pathology, Yonsei University, Seoul 03722, Republic of Korea; ^2^Department of Speech and Hearing Therapy, Catholic University of Pusan, Busan 46252, Republic of Korea; ^3^Neurocognitive Behavior Center, Seoul National University Bundang Hospital, Seongnam 13620, Republic of Korea; ^4^Department of Neurology, Seoul National University College of Medicine, Seoul 03080, Republic of Korea; ^5^Translational Medicine, Seoul National University College of Medicine, Seoul 03080, Republic of Korea; ^6^Department and Research Institute of Rehabilitation Medicine, Yonsei University College of Medicine, Seoul 03722, Republic of Korea

## Abstract

The aims of this study were to investigate concrete and abstract word definition ability (1) between patients with amnestic mild cognitive impairment (aMCI) and normal adults and (2) between the aMCI subtypes (i.e., amnestic single-domain MCI and amnestic multidomain MCI; asMCI and amMCI) and normal controls. The 68 patients with aMCI (29 asMCI and 39 amMCI) and 93 age- and education-matched normal adults performed word definition tasks composed of five concrete (e.g., train) and five abstract nouns (e.g., jealousy). Task performances were analyzed on total score, number of core meanings, and number of supplementary meanings. The results were as follows. First, the aMCI patients scored significantly poorer than the normal controls in only abstract word definition. Second, both subtypes of aMCI performed worse than the controls in only abstract word definition. In conclusion, a definition task of abstract rather than concrete concepts may provide richer information to show semantic impairment of aMCI.

## 1. Introduction

Mild cognitive impairment (MCI) is characterized by multifarious changes in linguistic areas as well as in cognitive domains. The most prominent characteristic of MCI is impairment in semantic knowledge in contrast with relatively well preserved phonological, morphological, and syntactic knowledge [1]. Previous studies of MCI have mainly focused on semantic aspects, which were identified using diverse language tasks such as confrontation naming [2, 3], verbal fluency [4, 5], and discourse [6, 7]. The confrontation naming tasks for patients with MCI have shown conflicting results, with one study demonstrating a difficulty with naming [1], while another showed no significant difference in naming ability compared with normal controls [2]. Likewise, the verbal fluency tasks had different outcomes due to the task types (e.g., categorical fluency, letter fluency) and the MCI experimental group characteristics [4, 5]. Both the confrontation naming and verbal fluency tasks examine semantic retrieval ability at the word level [6] and are therefore limited for demonstrating subtle changes in cognition and language in patients with MCI.

Discourse tasks, which demand more comprehensive and natural linguistic capacity, have been implemented to compensate for this limitation. Patients with MCI were shown to produce inadequate information and a high proportion of empty utterances when presented with such tasks [6, 7]. There are many types of discourse including narrative, procedural, expository, conversational, and descriptive. Different types and topics of discourse might generate diverse findings. In addition, discourse analysis is more time consuming than other language tasks and so may not be as well received for use in clinical settings.

To go elaborately into an examination of semantic characteristics of MCI, word definition tasks can be utilized. In word definition tasks, describing the meanings of words, the subjects' response is analyzed both in quantitative (i.e., numbers of semantic features produced for word definition) and qualitative (i.e., whether the semantic features are core or supplementary meanings) aspects [8]. The definition tasks, therefore, give a lot of information about the level of subjects' semantic processing. On the other hand, other semantic tasks require the subjects to generate the target words for their final response and consequently provide little information about the level of semantic processing achieved by them.

In spite of usefulness of word definition tasks for demonstrating subtle semantic degeneration, there are not many previous studies utilizing the tasks. Moreover, the researchers have most often carried on the tasks composed of only concrete words for various subject groups such as AD patients [9–11], normal elderly [12], children with language disorders [13], and normal children [14]. Concrete nouns are the names of tangible objects that one can experience or perceive with one's senses, which are the opposite of abstract nouns [15]. The conceptual knowledge for concrete and abstract nouns is stored in qualitatively different representational frameworks. Concrete concepts are supported in “categorical” frameworks, which have a close connection to super- and subordinate words in the same category. In contrast, abstract conceptualization involves integrated information in the “associative” framework. The associated features of abstract concepts have a horizontally spreading network [16]. Apart from the different representational frameworks, concrete and abstract concepts have different neural correlates. Based on neuroimaging studies, processing of high-level visual information of concrete words involves the left ventral temporal lobe whereas a high demand for semantic retrieval processing of abstract knowledge relies on the left inferior prefrontal lobe [17]. Finally, the development of definitions of concrete nouns takes place from preschool age, but that of abstract nouns is a gradual process from school age through adulthood, occurring over a relatively long period [18, 19]. Consequently, these distinctive characteristics of concrete and abstract concepts could be differentially impaired through cognitive and linguistic declines with neurological diseases.

To the best of our knowledge, there is no study on semantic processing of both concrete and abstract concepts for MCI. Because MCI deteriorate into AD with a high proportion, we might be allowed to some insights on differential impairment in concrete and abstract semantics through the research for AD. One study revealed that AD patients recalled more words with high imageability (i.e., concrete words) rather than those with low imageability (i.e., abstract words) in an immediate serial recall task. In addition, this deficit was accompanied with an abnormal proportion of phonological errors in the abstract word condition. In the synonym judgment task, AD patients also displayed selective impairment in abstract words comparing with concrete words [20]. Other researches have shown that AD patients have greater difficulties with abstract words than with concrete words in naming or semantic comprehension tasks [21–24]. Generally, the examinations of concrete and abstract knowledge for patients with AD have demonstrated relatively preserved concrete word processing in contrast with selective impairment in abstract concepts.

MCI is considered to be the intermediate stage from normal aging to dementia. Among the MCI subtypes, amnestic mild cognitive impairment (aMCI) is characterized as early-stage memory impairment and naming difficulty, and a large proportion of aMCI patients progress to Alzheimer's disease [25]. Recently, the determination of diagnosis subtypes such as amnestic single-domain MCI (asMCI) and amnestic multidomain MCI (amMCI) has become an important issue [26].

Thus, the purposes of this study were (1) to compare word definition ability (i.e., total score, number of core meanings, and number of supplementary meanings) between patients with aMCI and normal controls and (2) to identify any differences in word definition ability of aMCI patients with asMCI and amMCI subtypes compared to normal elderly.

## 2. Methods

### 2.1. Participants

This study included 68 patients with aMCI and 93 normal elderly patients. The aMCI patients were diagnosed by a neurologist according to the criteria proposed by Petersen et al.: (i) memory complaint usually corroborated by an informant; (ii) objective memory impairment for age; (iii) essentially preserved general cognitive function; (iv) largely intact functional activities; (v) being not demented [25]. All patients underwent neuropsychological tests using a standardized neuropsychological battery, called the Seoul Neuropsychological Screening Battery (SNSB) [27]. This battery covered tests for attention, language, praxis, the four symptoms of Gerstmann syndrome, visuoconstructive function, verbal and visual memory, and frontal/executive function.

On the basis of the profile of the neuropsychological tests, the aMCI patients were classified into two subtypes: 29 amnestic single-domain MCI (asMCI) patients with isolated memory dysfunction and 39 amnestic multidomain MCI (amMCI) patients with memory and other cognitive deficits.

Normal participants had no history of neurological or psychological disorders. They had adequate vision and hearing to perform the tasks. All participants scored within the normal range on the Korean version of the Mini-Mental State Examination (K-MMSE) [28] and had no symptoms of depression on the Geriatric Depression Scale Short Form-Korean Version (GDSSF-K) [29].

The demographic characteristics of aMCI patients and healthy controls are shown in Table 1. Independent *t*-tests revealed no significant differences between aMCI patients and normal controls with regard to age (*t* = 0.149, *p* > 0.05), sex (*χ* ^2^ = 0.221, *p* > 0.05), or years of education (*t* = −1.834, *p* > 0.05). One-way ANOVA showed statistical differences among asMCI, amMCI, and normal controls with regard to years of education (*F* = 4.495, *p* < 0.05) and MMSE scores (*F* = 5.068, *p* < 0.01) but not age (*F* = 0.069, *p* > 0.05) or sex (*χ* ^2^ = 0.299, *p* > 0.05). The neuropsychological profile of the patients is also displayed in Table 2.

We obtained informed consent from all participants, and the study was approved by the Institutional Review Boards of Seoul National University Bundang Hospital (IRB#: B-1402-240-106) for the patients with aMCI and of Severance Hospital (IRB#: 1-2011-0061) for normal elderly controls.

### 2.2. Materials: Word Definition Task

We developed the word definition task to be composed of five concrete and five abstract nouns. The five concrete nouns (i.e., “watermelon,” “rabbit,” “train,” “electric fan,” and “pharmacy”) were determined on the basis of semantic categories [30], definition categories [31], living and nonliving things [11], and imageability [9]. The representative semantic categories included vegetables, animals, transportations, electrical appliances, and places. Each word was associated with three or four definition categories (i.e., perceptual, functional, relational, and categorical). The relational and categorical definition categories were significantly less used by the patients with dementia compared to the normal controls, but the perceptual definition category showed no group differences [10]. Words prominent in only the perceptual definition category might not show age-related or neurological deterioration in word definition ability. In the next step, two words representing living things were chosen from the “animals” and “vegetables” categories. Two words representing man-made artifacts were also selected from the “transportations” and “electrical appliances” categories. The brain-damaged patients showed more severe impairment in the processing of living things than in the processing of nonliving things [32, 33]. As living things have the characteristics of strong structural similarity and high visual complexity, the patients had difficulty processing such features [34]. Finally, a less imageable word was added to the concrete word list. Linguistic information is processed in the left hemisphere and visual-perceptual information in the right hemisphere. Therefore, words with high imageability are associated with both hemispheres. On the contrary, the left hemisphere is mainly activated when processing abstract concepts [35]. Due to the absence of standardized data on imageability, 20 normal adults (mean age: 27.03, mean education years: 15.13) rated the imageability of each word based on a 5-point Likert scale, through the questionnaire (“How imageable is this word?”).

The five abstract words (i.e., “picnic,” “jealousy,” “music,” “friendship,” and “joke”) were selected after considering semantic categories, clarity of semantic features, and abstractness. After investigating abstract words list employed in the previous studies [19, 36, 37] and considering semantic categories of abstract concepts [38–40], we classified five semantic categories: emotion (e.g., happiness), traits (e.g., courage), social relation (e.g., friendship), mental state (e.g., consciousness), and action (e.g., performance). As abstract concepts do not directly correspond with entities in the physical world, as do concrete concepts, specific primary meanings are rarely fixed in abstract semantics [41]. As it is more difficult to define abstract nouns than concrete nouns, abstract concepts with clear semantic features were chosen for easier definition. Then, various levels of imageability were considered. The imageability of abstract nouns was investigated in the same way as with concrete nouns.

### 2.3. Procedures

After the participants filled out the demographic information and health state questionnaire, they completed the K-MMSE. For the word definition task, the participants were presented with concrete and abstract words in a random order on a computer screen. The examiner gave instructions to “Tell me what [sample word] is. Give me as much information about the word as possible.” Thirty seconds were allowed for participants to define each word. Within 30 seconds, the instruction could be repeated when the participants produced no response or few utterances. The semantic features of each definition were classified into core meanings and supplementary meanings.

To score the subjects' responses to the word definition task, the criteria of core and supplementary meanings should be determined by priority. In this study, “analysis of range” was applied to provide objective and valid criteria for core and supplementary meanings. “Analysis of range” has been generally utilized in the vocabulary selection process, and it involves how many times a word was repeatedly selected in the previous studies and references. The high-range words are considered very important and the low-range words relatively less important [42]. In this study, analysis of range was applied by extracting the high-range semantic features from the subjects' utterance samples.

First, 100 normal elderly participants were made to perform a word definition task. Each semantic feature was extracted from a word definition. The range was determined by identifying how many times the semantic feature appeared in the 100 elderly participants' word definitions. A higher range among the subjects was taken to mean that the semantic feature more prominently occurs when describing a word meaning than those with a low range. Consequently, the semantic features produced by more than ten out of the 100 elderly participants for the word definition task were determined as core meanings. Each core meaning was assigned two points and each supplementary meaning was assigned one point. The points were summed to produce the total score of the word definition task. The examples of word definition task scoring appear in Table 3. All the responses were recorded using a Conic digital voice recorder S-10.

### 2.4. Analysis

#### 2.4.1. Reliability Analysis

Interjudge reliability of coding for core and supplementary meanings was calculated by two researchers. One rater was the first author of this study, and the other rater was a doctoral graduate student in speech and language pathology. A total of 160 definitions (10% of all responses) were randomly selected. Interjudge agreement was 93%.

#### 2.4.2. Data Analysis

The results were first analyzed to compare aMCI patients and normal controls. The total word definition, number of core meanings, and number of supplementary meanings for concrete noun scores were analyzed. The same analyses were performed for abstract nouns. An independent *t*-test was performed to confirm the differences between two groups whose age, sex, and years of education were matched.

Second, asMCI, amMCI, and normal elderly patients were compared to investigate any differences in total score, number of core meanings, and number of supplementary meanings. We performed one-way analysis of covariance (ANCOVA) after adjusting for years of education and MMSE score. *p* values < 0.05 were considered statistically significant. Statistical analyses were carried out using SPSS 19.0 for Windows.

## 3. Results

### 3.1. Comparison of Word Definition Abilities between Patients with Amnestic Mild Cognitive Impairment and Normal Controls

For the performances on concrete word definition task between aMCI patients and normal controls (Table 4), the *t*-test showed no statistical differences in total score (*t* = 1.818, *p* > 0.05), number of core meanings (*t* = 1.545, *p* > 0.05), or number of supplementary meanings (*t* = 1.466, *p* > 0.05). However, for abstract nouns between two groups, the *t*-test showed statistical differences in total score (*t* = 2.353, *p* < 0.05) and number of supplementary meanings (*t* = 3.526, *p* < 0.01) but not in number of core meanings (*t* = 1.276, *p* > 0.05).

### 3.2. Comparison of Word Definition Abilities among Patients with Amnestic Single-Domain MCI, Amnestic Multidomain MCI, and Normal Controls

After adjusting for years of education and MMSE score, total score, number of core meanings, and number of supplementary meanings were compared among the three groups. The analysis of ANCOVA revealed no differences with regard to concrete nouns. However, the ANCOVA showed a significant difference in number of supplementary meanings of abstract nouns (*p* < 0.01) but not in total score or number of core meanings. Post hoc analyses indicated that all three groups were different with regard to number of supplementary meanings (*p* < 0.001) as shown in Table 5.

## 4. Discussion

Contextual information can be specified to process traits of “abstractness,” because abstract semantics is learned via use in sentences and association with other concepts [33]. A specific primary meaning is rarely fixed in the processing of abstract semantics because it does not correspond directly with entities in the physical world, as do concrete concepts [41]. In the present study, aMCI patients demonstrated a significant deficit in abstract noun definition tasks compared to normal elderly individuals. Additionally, both aMCI subtypes (amnestic single-domain MCI and amnestic multidomain MCI) had difficulty producing sufficient semantic features of abstract words.

There are at least three hypotheses to account for the semantic impairment in abstract concepts identified in aMCI patients. First, the impairment is based on the “concreteness effect.” Concrete concepts are more easily processed than abstract concepts. The Paivio's dual-code perspective [35] explains that concrete concepts rely on both language and sensorimotor information (i.e., verbal and nonverbal code), whereas abstract concepts are supported only by language (i.e., verbal code). In addition to the dual-code theory, concrete words have more related contextual information than abstract words [43], and they are described by a greater number of semantic features [44]. All of these explanations support the idea that concrete concepts take advantage of semantic processing than do abstract concepts. This explains the compatible performance on concrete noun definition tasks for aMCI individuals compared to the normal controls.

Second, there are different representational frameworks between abstract and concrete concepts. It has recently been proposed that abstract concepts are supported by an “associative” neural network and that abstract concepts are semantically connected with other associated knowledge in a horizontally arranged network. In contrast, concrete concepts are organized by category and have semantic features in super- and subordinate relations [17, 45]. According to the “spreading activation model” [46], the distances between concepts are differentiated by categorical relation, typicality, and the associated degrees of other concepts. A semantically long distance prevents rapid and strong activation through word processing. This semantically spreading integration occurs in the prefrontal regions [47] that are vulnerable to MCI. Therefore, patients with MCI have difficulty with semantically divergent activations, only processing primary meanings that have a close distance to a target word. As a result, only a partial specification of word meanings can be achieved, producing fewer supplementary meanings of an abstract word.

The third hypothesis to account for selective impairment in abstract word definition is that concrete and abstract concepts are different in preponderant semantic information. Affective information plays a greater role for abstract concepts, while sensory-motor information is more prominent for concrete concepts. Some researchers reported that abstract words have an advantage on processing semantic knowledge due to their greater affective associations [48, 49]. However, strong affective valence of abstract concepts might hinder proper description of word meanings and allow individuals to focus on their personal feelings and experiences related to the target word. For example, while defining the word of “music,” the word evoked abnormally strong positive or negative emotion. The patients could not generate appropriate semantic features but started to talk about their favorite music and singer and even ended in singing or crying.

Although aMCI patients exhibited abstract concept deficits, they demonstrated comparable performance levels in concrete noun definition tasks compared with the normal elderly group. These findings did not replicate those of the previous study that implemented a concrete word definition task for patients with MCI [50]. In Lim et al.'s study, patients with MCI revealed significantly lower scores than the control in a 22-concrete word definition task. However, the differences in findings of the current study and Lim et al.'s may be attributable to the following three reasons: first one could be due to methodological differences such as the numbers of subjects (e.g., 68 versus 8) that were employed in the studies. Second reason could be ascribed to MCI subtype specification. The previous study did not classify MCI subtypes. However, in this current study, we divided the subtypes of MCI because each subtype could be heterogeneously distinctive in nature [25]. Specifically, the patients that were classified as aMCI have demonstrated greater progression to Alzheimer's disease and are considered to be a significant pathological group. The third reason for the difference was that they employed different scoring methods. Whereas the previous study used a 3-point equal-interval scoring method (e.g., 0, 1, and 2 points), we adopted total scores (sum of core and supplementary meanings) which may be more multidimensional and, therefore, more sensitive to subtle pathological group differences [51].

Intactness in core and superordinate knowledge in word concepts observed in aMCI has not been found in AD patients. The previous studies of AD patients demonstrated a qualitatively weaker definition ability and fewer primary features of target words compared with the normal [9, 10]. In this study, distinction was made between aMCI and AD patients which may serve as a linguistic-behavioral marker for differentiating the two.

The results of the study are noteworthy because it is the first study to utilize abstract nouns in word definition task to examine semantic knowledge impairment in patients with aMCI. Abstract words (e.g., “sensible,” “confident”) in registration and recall tasks have been included in the recently developed Mini-Mental State Examination, 2nd edition (MMSE-2) [52]. The MMSE-2 was developed to provide finer discrimination as a reliable cognitive screening measure because the previous version of the MMSE has been known to be less sensitive for detecting patients with MCI and in the early stages of dementia [53].

In conclusion, a definition task of abstract rather than concrete concepts may provide richer information to show semantic impairment of aMCI. Semantic deficits of aMCI may often go unnoticed due to their mild nature, which in turn requires highly sensitive linguistic tasks for detecting semantic deficits. Future study is needed to confirm the process of semantic knowledge deteriorated from aMCI to various stages of AD.

## Figures and Tables

**Table 1:** Participant characteristics.

Group	Subgroup	*N*	Age (years)	Sex (M : F)	Education (years)	K-MMSE
aMCI		68	73.24 ± 8.87	29 : 39	12.63 ± 4.26	26.17 ± 1.72
	asMCI	29	73.00 ± 8.86	13 : 16	12.48 ± 4.14	26.85 ± 2.93
	amMCI	39	73.41 ± 8.99	16 : 23	13.62 ± 4.10	26.00 ± 1.96

Normal		93	72.80 ± 8.46	36 : 57	11.51 ± 3.43	27.33 ± 2.02

Data are presented as mean ± SD. aMCI: amnestic mild cognitive impairment. asMCI: amnestic single-domain mild cognitive impairment. amMCI: amnestic multidomain mild cognitive impairment. K-MMSE: Korean version of the Mini-Mental State Examination.

**Table 2:** Neuropsychological profile of patients.

	aMCI (total)	asMCI	amMCI
	(*n* = 68)	(*n* = 29)	(*n* = 39)
Attention			
Digit span forward test	5.76 ± 1.30	5.78 ± 1.48	5.74 ± 1.19
Digit span backward test	3.85 ± 1.47	3.89 ± 1.22	3.82 ± 1.64
Language function			
K-BNT	10.06 ± 1.46	10.78 ± 2.21	9.56 ± 2.52
Visuospatial function			
RCFT copy	30.50 ± 4.12	32.28 ± 2.63	29.27 ± 4.53
Memory function			
SVLT immediate recall	16.41 ± 4.17	17.74 ± 3.93	15.49 ± 4.13
SVLT delayed recall	2.80 ± 2.66	3.48 ± 2.86	2.33 ± 2.44
SVLT recognition	6.47 ± 2.41	6.70 ± 2.69	6.31 ± 2.23
RCFT immediate recall	9.15 ± 5.85	10.20 ± 5.77	8.42 ± 5.86
RCFT delayed recall	8.94 ± 6.07	10.04 ± 5.92	8.18 ± 6.13
RCFT recognition	6.48 ± 1.72	6.72 ± 1.80	6.31 ± 1.67
Frontal function			
Semantic COWAT (animal)	13.20 ± 4.13	14.37 ± 3.95	12.38 ± 4.10
Phonemic COWAT	7.11 ± 3.36	7.04 ± 3.14	7.15 ± 3.54

Data are presented as mean ± SD. aMCI: amnestic mild cognitive impairment. asMCI: amnestic single-domain mild cognitive impairment. amMCI: amnestic multidomain mild cognitive impairment. K-BNT: Korean version of Boston Naming Test. RCFT: Rey Complex Figure Test. SVLT: Seoul Verbal Learning Test (three free recall trials of 12 words and a 20-minute delayed recall trial and recognition test). COWAT: Controlled Oral Word Association Test.

**Table 3:** Examples of word definition task scoring.

Word	Participant definition	Core meanings	Supplementary meanings	Total score
Watermelon (concrete noun)	We usually *eat it in summer*. It is **cool**. *A fruit *that we eat in a hot summer because it is **juicy**.	(1) Eat in summer (2) A fruit	(1) Cool (2) Juicy	2 × 2 + 1 × 2 = 6

Joke (abstract noun)	A thing to *make others laugh*, particularly *among intimates*. **A coarse joke is liable to be misunderstood**.	(1) Make others laugh (2) Among intimates	Liable to be misunderstood	2 × 2 + 1 × 1 = 5

**Table 4:** Word definition task performance for aMCI patients and normal controls.

		aMCI patients	Normal controls	*p* value
Concrete word	Total score	27.61 ± 7.20	30.34 ± 8.91	.72
	No. of core meanings	11.55 ± 3.15	12.52 ± 3.57	.125
	No. of supplementary meanings	4.50 ± 2.65	5.31 ± 3.26	.145

Abstract word	Total score	19.45 ± 5.87	22.23 ± 6.85	.020^*∗*^
	No. of core meanings	8.46 ± 2.80	9.15 ± 2.97	.204
	No. of supplementary meanings	2.52 ± 2.02	3.94 ± 2.31	.001^*∗∗*^

Data are presented as mean ± SD. aMCI: amnestic mild cognitive impairment. No.: number.

^*∗*^ *p* < .05,^*∗∗*^ *p* < .01.

**Table 5:** Word definition task performance for asMCI, amMCI, and normal controls.

		asMCI patients	amMCI patients	Normal controls	*p* value
Concrete word	Total score	29.37 ± 6.27	29.28 ± 8.61	28.61 ± 8.92	.948
	No. of core meanings	12.63 ± 2.65	11.92 ± 3.55	11.56 ± 3.61	.383
	No. of supplementary meanings	4.11 ± 2.50	5.44 ± 3.08	5.50 ± 3.59	.103

Abstract word	Total score	21.17 ± 8.61	20.85 ± 6.85	20.91 ± 7.21	.975
	No. of core meanings	8.85 ± 2.84	9.37 ± 3.16	8.42 ± 3.27	.466
	No. of supplementary meanings	3.49 ± 2.40	2.11 ± 1.57	4.07 ± 2.73	.001^*∗∗*^

Data are presented as mean ± SD. asMCI: amnestic single-domain mild cognitive impairment. amMCI: amnestic multidomain mild cognitive impairment. No.: number.

^*∗∗*^ *p* < .01.
